# Supplementary material for: Exploring the performance and explainability of fine-tuned BERT models for neuroradiology protocol assignment
Source: BMC Med Inform Decis Mak. 2024 Feb 7;24:40. doi: 10.1186/s12911-024-02444-z (PMC10848624; doi:10.1186/s12911-024-02444-z)
Supplement: Supplementary file 1 — Additional file 1: Supplemental. Word Importance [file 12911_2024_2444_MOESM1_ESM.docx]

**SUPPLEMENTAL**

1. *Word Importance*

Integrated gradients exploit the gradient information of the model by integrating first-order derivatives. This method does not require the model to be differentiable or smooth, making it particularly suitable for large and complex models such as Transformers. We use integrated gradients to accurately estimate the importance of individual words within an input sentence.

The integrated gradients method can be formally defined as follows: let x be the input sentence,

represented as a (*x*_1_*, ..., x_m_*), and let *x^′^* be a “blank” baseline input. We have a trained model *F* , and *F* (*x*)*_n_* is the output of the model at time step n. The contribution of the *mth* word in *x* to the prediction of *F* (*x*)*_n_* can be calculated by taking the integral of gradients along the straight line path from *x^′^* to the input *x*. In other words, we are measuring how much the prediction at time step *n* changes as we move from the baseline input *x^′^* to the actual input *x*, and specifically how much the *mth* word in *x* contributes to this change.

The word importance value of each word in the input is calculated by summing the scalar attributions across the dimensions of the input embeddings. A positive attribution value indicates that the word contributed to the prediction made by the model, while a negative attribution value indicates that it opposed the prediction. In cases of the BERT model, which uses sub-word tokenization to divide rare words into smaller pieces, we can obtain word-level attributions that are more understandable to humans by taking the sub-word with the highest absolute attribution value as the attribution for the entire word.
